# Ultrasound-Facilitated, Catheter-Directed Thrombolysis for Acute Pulmonary Embolism

**DOI:** 10.7759/cureus.57345

**Published:** 2024-03-31

**Authors:** Ahmed A Elheet, Amr F Elhadidy, Mohamad H Farrag, Mohamed A Mahmoud, Ayman A Ibrahim, Ali M AlAbdali, Hameedullah Kazim, Mohammed N Elganainy

**Affiliations:** 1 Cardiovascular Disease, Mahalla Cardiac Center, Tanta, EGY; 2 Cardiovascular Disease, Al Hada Armed Forces Hospital, Taif, SAU; 3 Cardiology, Al Hada Armed Force Hospital, Taif, SAU; 4 Cardiovascular Medicine, Al Hada Armed Force Hospital, Taif, SAU; 5 Cardiovascular Disease, Al Hada Armed Force Hospital, Taif, SAU

**Keywords:** major bleeding events, rv/lv diameter ratio, cdt, uscdt, acute pulmonary embolism

## Abstract

Background: Acute pulmonary embolism (APE) poses a significant risk to patient health, with treatment options varying in efficacy and safety. Ultrasound-facilitated catheter-directed thrombolysis (USCDT) has emerged as a potential alternative to conventional catheter-directed thrombolysis (CDT) for patients with intermediate to high-risk APE. This study aimed to compare the efficacy and safety of USCDT versus conventional CDT in patients with intermediate to high-risk APE.

Methods: This observational retrospective study was conducted at the Armed Forces Hospital, Al-Hada, Taif, the Kingdom of Saudi Arabia (KSA), on 135 patients diagnosed with APE and treated with either USCDT or CDT (58 underwent CDT, while 77 underwent USCDT). The primary efficacy outcome was the change in the right ventricle to the left ventricle (RV/LV) diameter ratio. Secondary outcomes included changes in pulmonary artery systolic pressure and the Miller angiographic obstruction index score. Safety outcomes focused on major bleeding events.

Results: Both USCDT and CDT significantly reduced RV/LV diameter ratio (from 1.35 ± 0.14 to 1.05 ± 0.17, P < 0.001) and systolic pulmonary artery pressure (SPAP) (from 55 ± 7 mmHg to 38 ± 7 mmHg, P < 0.001) at 48- and 12-hours post-procedure, respectively, with no significant differences between treatments. However, USCDT was associated with a significantly lower rate of major bleeding events compared to CDT (0% vs. 3.4%, P = 0.008). Multivariate logistic regression analysis revealed that USCDT was associated with a 71.9% risk reduction of bleeding (OR = 0.281, 95% CI = 0.126 - 0.627, P = 0.002).

Conclusions: USCDT is a safe and effective alternative to CDT for the treatment of intermediate to high-risk APE, as it significantly reduces the risk of major bleeding.

## Introduction

Acute pulmonary embolism (APE) is a critical health issue that significantly contributes to global morbidity and mortality, with post-pulmonary embolism syndrome affecting up to 50% of survivors and altering their quality of life [1]. This condition can be stratified into different risk categories based on clinical, imaging, and laboratory data, which are essential for guiding treatment strategies [2]. High-risk (massive) and intermediate-risk (submassive) APE are particularly concerning due to their substantial contribution to patient death and morbidity [3]. While systemic anticoagulation serves as the cornerstone of initial therapy [4], the management of APE, especially in high and intermediate-risk groups, presents complex challenges.

High-risk APE, which constitutes less than 10% of cases, demands urgent reperfusion therapy to avert imminent mortality [5]. Although systemic thrombolysis is recommended for these patients, the actual application is limited due to the high risk of haemorrhagic complications or the presence of contraindications in a significant subset of patients [6]. Furthermore, about 8% of those treated with systemic thrombolysis experience delayed therapeutic responses. As an alternative, surgical pulmonary embolectomy offers another reperfusion strategy but suffers from limited availability and high procedural risk [7].

Intermediate-risk APE, accounting for 45 to 65% of incidents, poses a unique treatment dilemma. These patients face a baseline short-term mortality rate of around 3%, which does not justify the risk of systemic thrombolysis [8]. However, a subset within this group has a mortality rate of approximately 12%, underscoring the inadequacy of anticoagulation alone and highlighting the need for more aggressive reperfusion strategies in selected patients [1]. This scenario emphasizes the potential of catheter-directed therapies, such as catheter embolectomy and catheter-directed thrombolysis (CDT), as either primary treatments or alternatives when systemic thrombolysis is contraindicated or fails [9]. CDT, by delivering a fraction of the systemic dose directly to the thrombus, significantly reduces the risk of bleeding complications, offering a safer option for many patients. Moreover, for those unable to undergo thrombolysis, mechanical thrombus removal via catheter embolectomy presents a viable intervention [10].

Amidst these therapeutic options, the development of ultrasound-facilitated catheter-directed thrombolysis (USCDT) introduces a novel approach that combines the mechanical action of ultrasound with the pharmacological benefits of thrombolytic agents. This innovative treatment aims to enhance the dissolution of pulmonary emboli, showing superior outcomes compared to traditional methods.

Despite the promising potential of USCDT, empirical evidence comparing its efficacy and safety directly against traditional CDT, particularly for intermediate to high-risk APE patients, remains scarce. Therefore, the objective of this study was to evaluate the effectiveness and safety of USCDT with conventional CDT in patients diagnosed with intermediate to high-risk APE.

## Materials and methods

Study design and setting

This single-center, observational retrospective study enrolled 135 consecutive patients admitted with APE and were treated by USCDT or conventional CDT at the catheterization laboratory in Armed Forces Hospital, Al-Hada, Taif, KSA, for 4.5 years (from January 2018 to June 2022). The study included patients aged 18 to 75 years who present with intermediate-risk APE, characterized by pulmonary embolism (PE) symptoms for less than 14 days, normal SBP (>90 mm Hg), and evidence of right ventricle (RV) compromise. It also included selected patients with high-risk APE, defined by PE symptoms for less than 14 days, hypotension (SBP <90 mm Hg) that responds well to supportive treatment without signs of end-organ hypoperfusion, and evidence of RV compromise.

Eligible participants were required to exhibit a right ventricle to the left ventricle (RV to LV) diameter ratio exceeding 0.9, as determined by chest CTA, and a proximal PE in at least one main or proximal lobar artery. Exclusion criteria were PE symptom duration of >14 days, allergy or hypersensitivity to heparin, severe contrast allergy to iodinated contrast, known right-to-left cardiac shunt, large (>10 mm) right atrial or RV thrombus, and hemodynamic decompensation. Other exclusions encompass recent stroke, head trauma, major surgery, gastrointestinal bleeding, active bleeding or bleeding disorders, recent thrombolytic therapy, prolonged cardiopulmonary resuscitation, severe renal dysfunction (eGFR less than 15ml/min/1.73m^2^), active cancer with a life expectancy of <6 months, pregnancy or lactation, and an inability to comply with study assessments.

Study protocol and methods

Between January 2018 and June 2022, 135 patients with APE were admitted to undergo treatment involving unfractionated heparin (UFH) combined with a treatment course of 12 mg recombinant tissue plasminogen activator (rtPA) administered over 12 hours for each affected lung, following either a USCDT or conventional CDT protocol.

This intervention was carried out adhering to the established methods for USCDT or CDT, executed by a skilled specialist in Interventional Cardiology. For venous access, the common femoral vein was accessed under ultrasound guidance in all cases, utilizing 6-Fr sheaths. The 5.2-F catheter used for infusion was designed with three channels: one for the ultrasound wire, one for administering the medication, and one for circulating coolant (sterile saline). The Ekos catheter's insertion was precisely directed using fluoroscopy. In cases where the pulmonary embolism was unilateral, affecting a single main or proximal lobar artery, a single catheter was placed within the affected artery (patients in this scenario received a halved dose compared to those with bilateral embolisms). For bilateral pulmonary embolisms involving main or proximal lobar arteries, two catheters were utilized, with one inserted into each affected artery. The therapeutic regimen commenced with the administration of rtPA and saline coolant at a rate of 30 ml/h for each catheter. An invasive hemodynamic evaluation was conducted 12 hours following the start of fibrinolysis to assess the outcomes of the procedure.

Prior to initiating the recruitment process, standard transthoracic echocardiograms paired with ECG recordings were collected as baseline data. Given that an RV/LV diameter ratio greater than 0.9 was a key requirement for participation and the primary goal was to track changes in this ratio from the initial assessment to the 24-hour mark, special emphasis was placed on acquiring a minimum of four clear cine loops from the apical four-chamber perspective to accurately determine the RV/LV ratio. Echocardiographic records from these evaluations at the onset, after 24 hours, and at 90 days were forwarded to the principal laboratory for analysis.

The echocardiographic analysis, particularly from the apical four-chamber angle, covered measurements such as the sub-annular end-diastolic RV/LV ratio and the movement of the tricuspid annular plane during systole. Additionally, right ventricular systolic function was assessed and categorized as none, mild, moderate, or severe based on observations from both the parasternal short-axis and apical four-chamber angles, while the smallest diameter of the intrahepatic segment of the inferior vena cava was measured using a subcostal approach.

Electronic versions of the initial and 48-hour follow-up chest CT scans were sent to the central CT laboratory. This facility verified the diagnosis of PE in all participants and analyzed the follow-up outcomes. Furthermore, this laboratory determined the pulmonary occlusion scores using The Miller index. This index includes an objective score for arterial blockage and a subjective evaluation score for the decrease in lung peripheral perfusion, with the highest possible score for blockage being 16 and for perfusion reduction being 18.

The maximum possible Miller index for each patient is 34. Any segmental emboli identified are assigned a score of 1 point, regardless of the extent of obstruction. Emboli located proximally to the segmental arteries receive a score that matches the number of segmental arteries branching off below them, which is in line with previously established anatomical segmentation.

Every patient was administered therapeutic anticoagulation following the deep vein thrombosis (DVT) guidelines, which include an initial dose of 80 units/kg as a bolus, followed by a continuous infusion of 18 units/kg/hour to maintain the Partial Thromboplastin Time (PTT) within a target range of 60-80 seconds. During the administration of thrombolytic therapy, the dosage of heparin was adjusted to a lower rate of 300 to 500 IU/hour. Once the drug delivery devices were removed, the site of insertion was directly pressed for a duration of 10 to 15 minutes to facilitate clotting. Anticoagulation therapy was resumed at its full therapeutic dosage 20 minutes after successful clot formation. The specific anticoagulation regimen, including its type and length, was prescribed by the overseeing physician post the USCDT or CDT treatment.

Furthermore, in many cases, should the thrombolytic treatment be deemed insufficient after roughly 12 hours, the medical practitioner might opt for an additional rheolytic thrombectomy, with or without further thrombolysis, depending largely on the extent of the remaining thrombus.

Clinical and laboratory data were reviewed for all selected groups, covering a range of variables including age, gender, history of thromboembolism (either deep vein thrombosis or pulmonary embolism), histories of renal, hepatic, or cancer diseases, and the duration of PE symptoms. This review also included routine baseline laboratory investigations, with a particular focus on cardiac troponin I (cTnI) and B-type natriuretic peptide (BNP). Follow-up after the pulmonary embolism event involved clinical visits and routine blood sampling at one and three months, during which a two-dimensional echocardiogram was obtained. Beyond the initial three-month period, regular monthly follow-ups were conducted, including clinical examinations and routine laboratory tests. Data collection was facilitated through a standard pre-catheterization sheet, approved by the cardiology department tailored for each patient.

Outcomes

This research assessed the effectiveness and safety of two treatment approaches by examining various outcomes. The primary measure of effectiveness was the variation in the ratio of the RV/LV diameter, calculated by the core laboratory using contrast-enhanced chest CT scans taken before the treatment and 48 hours after its commencement. Secondary measures of effectiveness included the alteration in the systolic pressure of the pulmonary artery, determined via right heart catheterization and transthoracic echocardiography at the 48-hour mark post-treatment, as well as the change in the score of the modified Miller angiographic obstruction index, which was assessed using contrast-enhanced chest CT scans at baseline and the 48-hour follow-up.

The main safety endpoint was the occurrence of significant bleeding events within the first 96 hours following the procedure, categorized based on the criteria set by the Global Utilization of Streptokinase and Tissue Plasminogen Activator for Occluded Coronary Arteries (GUSTO) guidelines [11], with all such incidents monitored during the patients' hospital stay.

Secondary safety outcomes investigated the incidence of symptomatic recurrent pulmonary embolism within 90 days after the procedure, mortality from any cause at the time of hospital discharge and within 30 days, and any complications related to the technical aspects of the procedure.

Statistical analysis

Data management and analysis were carried out with SPSS software, Version 28.0 (IBM Corp., Armonk, NY). The normal distribution of quantitative data was evaluated using the Kolmogorov-Smirnov test along with methods of direct visualization. Depending on their distribution, quantitative data were presented as means with standard deviations, while categorical data were expressed in counts and percentages. Comparisons of quantitative data across different procedure types were conducted using the independent t-test for continuous variables and the Chi-square or Fisher's exact test for categorical variables. For comparisons within groups, the paired t-test was utilized for analyses involving two time points, and repeated measures ANOVA was applied for comparisons across more than two time points. Multivariate logistic regression was employed to assess the risk of bleeding, from which odds ratios and 95% confidence intervals were derived. All statistical testing was two-sided, and a P-value of less than 0.05 was deemed to indicate statistical significance.

## Results

General and demographic characteristics

Of the 135 patients, 58 underwent CDT, while 77 underwent USCDT. No significant differences were observed between those who underwent CDT and those who underwent USCDT regarding age (P = 0.254), sex (P = 0.782), diabetes mellitus (P =0.819), hypertension (P = 0.528), BMI (P = 0.359), previous DVT (P = 0.819), previous PE (P = 0.676), contraceptive use (P = 0.862), previous stroke (P = 0.634), CKD (P = 0.715), autoimmune disease (P = 0.545), cancer (P = 0.715), Immobility within 30 days (P = 0.862), active infection within 30 days (P = 0.807), CHF (P = 0.730), symptoms < 14 days, DVT on US (P = 0.768), syncope (P = 0.940), hypotension (P = 0.193), hypoxemia < 93% (P = 0.972), tachycardia > 100 b/m (P = 0.163), elevated BNP (P = 0.974), and high hs troponin (P = 0.642) (Table 1).

**Table 1:** Demographic and general characteristics of CDT and USCDT groups SD: Standard deviation; CDT: Catheter-directed thrombolysis; USCDT: Ultrasound-facilitated catheter-directed thrombolysis (USCDT); BMI: Body mass index; DVT: Deep vein thrombosis; PE: Pulmonary embolism; CKD: Chronic kidney disease; CHF: Congestive heart failure; US: Ultrasound; BNP: B-type natriuretic peptide; hs troponin: High-sensitivity troponin.

Parameter		Total (n = 135)	CDT (n = 58)	USAT (n = 77)	P-value
Age (years)	Mean ±SD	59 ±10	58 ±10	60 ±10	0.254
Sex	------				
Males	n (%)	61 (45.2)	27 (46.6)	34 (44.2)	0.782
Females	n (%)	74 (54.8)	31 (53.4)	43 (55.8)	----
Diabetes mellitus	n (%)	29 (21.5)	13 (22.4)	16 (20.8)	0.819
Hypertension	n (%)	82 (60.7)	37 (63.8)	45 (58.4)	0.528
BMI	------				
18 - 24	n (%)	34 (25.2)	19 (32.8)	15 (19.5)	0.359
25 - 30	n (%)	33 (24.4)	12 (20.7)	21 (27.3)	----
31 - 35	n (%)	36 (26.7)	14 (24.1)	22 (28.6)	----
> 35	n (%)	32 (23.7)	13 (22.4)	19 (24.7)	----
Previous DVT	n (%)	29 (21.5)	13 (22.4)	16 (20.8)	0.819
Previous PE	n (%)	19 (14.1)	9 (15.5)	10 (13)	0.676
Contraceptive use	n (%)	11 (8.1)	5 (8.6)	6 (7.8)	0.862
Previous stroke	n (%)	4 (3)	1 (1.7)	3 (3.9)	0.634
CKD	n (%)	17 (12.6)	8 (13.8)	9 (11.7)	0.715
Autoimmune disease	n (%)	16 (11.9)	8 (13.8)	8 (10.4)	0.545
Cancer	n (%)	17 (12.6)	8 (13.8)	9 (11.7)	0.715
Immobility within 30 days	n (%)	11 (8.1)	5 (8.6)	6 (7.8)	0.862
Active infection within 30 days	n (%)	13 (9.6)	6 (10.3)	7 (9.1)	0.807
CHF	n (%)	13 (9.6)	5 (8.6)	8 (10.4)	0.730
Symptoms < 14 days	n (%)	135 (100)	58 (100)	77 (100)	----
DVT on US	n (%)	47 (34.8)	21 (36.2)	26 (33.8)	0.768
Syncope	n (%)	26 (19.3)	11 (19)	15 (19.5)	0.940
Hypotension	n (%)	30 (22.2)	16 (27.6)	14 (18.2)	0.193
Hypoxemia < 93%	n (%)	44 (32.6)	19 (32.8)	25 (32.5)	0.972
Tachycardia > 100 b/m	n (%)	67 (50)	25 (43.1)	42 (55.3)	0.163
Elevated BNP	n (%)	88 (65.7)	38 (65.5)	50 (65.8)	0.974
High hs troponin	n (%)	105 (77.8)	44 (75.9)	61 (79.2)	0.642

Baseline and follow-up echo, catheter, and CT findings

Baseline echo findings did not significantly differ between patients who underwent CDT and those who underwent USCDT, including tricuspid annular plane systolic excursion (TAPSE) (P = 0.754), RV/LV ratio (P = 0.770), and minimum inferior vena cava (IVC) diameter (P = 0.664). Regarding follow-up data, echo findings, including TAPSE, RV/LV ratio, and IVC diameter, were comparable between patients who underwent CDT and those who underwent USCDT at 24 hours (P = 0.853, 0.914, and 0.186, respectively) and at 90 days (P = 0.808, 0.548, and 0.085, respectively) (Table 2).

**Table 2:** Baseline and follow-up echo, catheter, and CT findings in CDT and USCDT groups *Significant P-value; † Significantly different from baseline; TAPSE: Tricuspid annular plane systolic excursion; RV: Right ventricle; LV: Left ventricle; IVC: Inferior vena cava; SPAP: Systolic pulmonary artery pressure; CT: Computed tomography; PA: Pulmonary artery.

Parameter		Total (n = 135)	CDT (n = 58)	USCDT (n = 77)	P-value
ECHO	--------				
TAPSE (mm)					
Baseline	Mean ±SD	15.95 ±1.4	15.99 ±1.39	15.91 ±1.42	0.754
At 24 hours	Mean ±SD	18.91 ±1.66	18.94 ±1.73†	18.88 ±1.62†	0.853
90 days	Mean ±SD	21.22 ±1.75	21.27 ±1.86†	21.19 ±1.67†	0.808
P-value	----		<0.001*	<0.001*	----
RV/LV ratio					
Baseline	Mean ±SD	1.35 ±0.14	1.35 ±0.13	1.35 ±0.14	0.77
At 24 hours	Mean ±SD	1.05 ±0.17	1.05 ±0.15†	1.05 ±0.19†	0.914
90 days	Mean ±SD	0.85 ±0.12	0.84 ±0.13†	0.86 ±0.12†	0.548
P-value	----		<0.001*	<0.001*	----
Minimum IVC diameter (mm)					
Baseline	Mean ±SD	17.31 ±2.28	17.41 ±2.16	17.24 ±2.37	0.664
At 24 hours	Mean ±SD	12.85 ±2.61	13.2 ±2.29†	12.6 ±2.8†	0.186
90 days	Mean ±SD	10.71 ±2.11	11.1 ±1.51†	10.44 ±2.43†	0.085
P-value	----		<0.001*	<0.001*	----
Catheter	----				
SPAP (mmHg)					
Baseline	Mean ±SD	55 ±7	54 ±6	55 ±7	0.746
At 12 hours	Mean ±SD	38 ±7	38 ±7†	38 ±6†	0.684
At 90 days	Mean ±SD	34 ±5	34 ±5†	34 ±4†	0.955
P-value	----		<0.001*	<0.001*	----
Cardiac index (L/m/m2)					
Baseline	Mean ±SD	2.58 ±0.39	2.6 ±0.42	2.57 ±0.36	0.658
At 12 hours	Mean ±SD	3.96 ±0.58	3.98 ±0.67	3.95 ±0.5	0.771
P-value			<0.001*	<0.001*	
CT	----				
RV/LV ratio					
Baseline	Mean ±SD	1.36 ±0.13	1.38 ±0.12	1.35 ±0.13	0.153
At 48 hours	Mean ±SD	1.05 ±0.17	1.05 ±0.15	1.05 ±0.19	0.884
P-value	----		<0.001*	<0.001*	----
Modified Miller index	----				
Baseline	Mean ±SD	21 ±2	21 ±2	21 ±2	0.699
At 48 hours	Mean ±SD	16 ±2	16 ±3	16 ±2	0.683
P-value	----		<0.001*	<0.001*	----
Location of thrombus					
Bilateral main PA	n (%)	39 (28.9)	14 (24.1)	25 (32.5)	0.517
Bilateral lower PA	n (%)	68 (50.4)	33 (56.9)	35 (45.5)	----
Unilateral upper	n (%)	15 (11.1)	5 (8.6)	10 (13)	----
Unilateral lower	n (%)	13 (9.6)	6 (10.3)	7 (9.1)	----

Additionally, baseline catheter findings did not significantly differ, including SPAP (P = 0.746) and cardiac index (P = 0.658). At follow-up, catheter findings did not significantly differ between CDT and USCDT groups at 12 hours, including SPAP (P = 0.684) and CI (P = 0.771). Moreover, at 90 days, no significant difference was observed regarding SPAP (P = 0.955) (Table 2). Furthermore, baseline CT findings were comparable between the groups, including RV/LV ratio (P = 0.153), modified Miller index (P = 0.699), and location of the thrombus (P = 0.517). Forty-eight-hour CT findings demonstrated no significant difference between CDT and USCDT groups, including RV/LV ratio (P = 0.884) and modified Miller index (P = 0.683) (Table 2).

Within each group, all echo parameters significantly differed at 24 hours and 90 days compared to baseline values. Regarding catheter parameters within CDT and USCDT groups, SPAP significantly differed at 12 hours and 90 days compared to baseline, and cardiac index significantly differed at 12 hours compared to baseline. Moreover, CT parameters, including RV/LV ratio and modified Miller index, significantly differed within each group at 48 hours compared to baseline (Table 2).

Procedural and post-procedural findings

**Figure 1:**
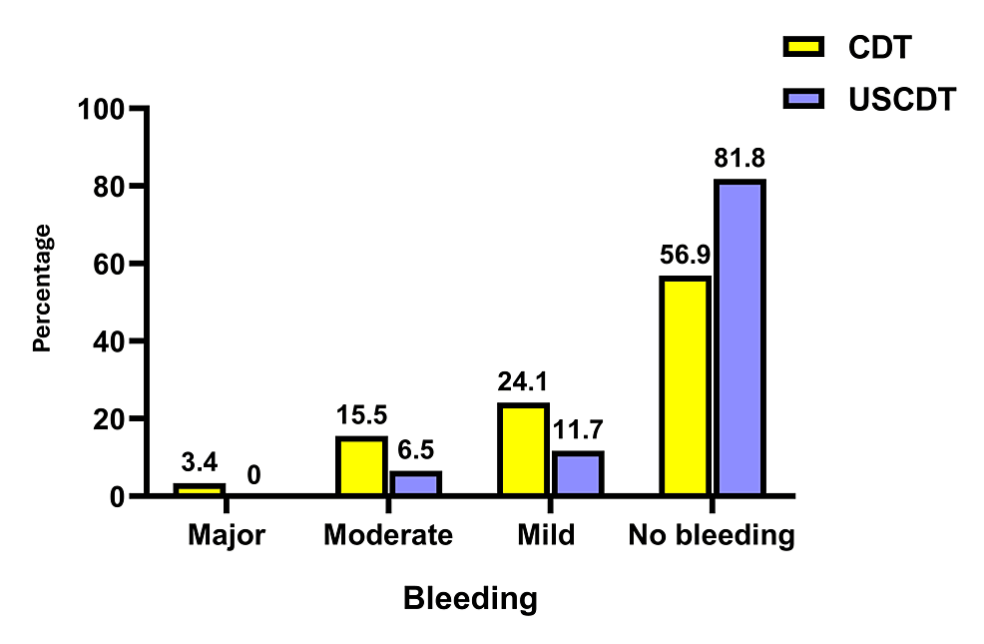
Bleeding in CDT and USCDT groups USCDT: Ultrasound-facilitated catheter-directed thrombolysis; CDT: Conventional catheter-directed thrombolysis.

Bleeding within 30 days significantly differed between patients who underwent CDT and those who underwent USCDT (P = 0.008), with major, moderate, and mild bleeding being higher in the CDT group (3.4%, 15.5%, and 24.1%, respectively) compared to the USCDT group (0%, 6.5%, and 11.7%, respectively) (Table 3 and Figure 1). No significant differences were observed regarding access site (P = 0.950), access sheath number (P = 0.491), TPA dose for 12 h (P = 0.211), number of devices and catheter (P = 0.176), length of stay (P = 0.643), in-hospital death (P = 1.0), 30-day mortality (P = 0.137), blood transfusion (P = 0.095), anaemia (P = 0.095), access site pseudoaneurysm (P = 0.430), access site aneurysm, hematoma (P = 0.278), haemoptysis (P = 0.077), haematuria (P = 0.364), recurrent PE (P = 1.0), and IVC filter (P = 0.430) (Table 3).

**Table 3:** Procedural and post-procedural findings in CDT and USCDT groups RT: Right; TPA: Tissue plasminogen activator; PE: Pulmonary embolism; IVC: Inferior vena cava; SD: Standard deviation.

Parameter		Total (n = 135)	CDT (n = 58)	USCDT (n = 77)	P-value
Access site					
RT femoral	n (%)	78 (57.8)	33 (56.9)	45 (58.4)	0.950
Left femoral	n (%)	40 (29.6)	18 (31)	22 (28.6)	----
Both	n (%)	17 (12.6)	7 (12.1)	10 (13)	----
Access sheath number					
Zero	n (%)	0 (0)	0 (0)	0 (0)	0.491
One	n (%)	115 (85.2)	48 (82.8)	67 (87)	----
Two	n (%)	20 (14.8)	10 (17.2)	10 (13)	----
TPA dose for 12 H					
Unilateral	n (%)	82 (61.2)	32 (55.2)	50 (65.8)	0.211
Bilateral	n (%)	52 (38.8)	26 (44.8)	26 (34.2)	----
Number of devices and catheters					
One	n (%)	45 (33.3)	23 (39.7)	22 (28.6)	0.176
Two	n (%)	90 (66.7)	35 (60.3)	55 (71.4)	
Length of stay	Mean ±SD	6 ±2	6 ±1	6 ±2	0.643
In-hospital death	n (%)	5 (3.7)	2 (3.4)	3 (3.9)	1
30-day mortality	n (%)	9 (6.7)	6 (10.3)	3 (3.9)	0.137
Bleeding within 30 days					
Major	n (%)	2 (1.5)	2 (3.4)	0 (0)	0.008*
Moderate	n (%)	14 (10.4)	9 (15.5)	5 (6.5)	
Mild	n (%)	23 (17)	14 (24.1)	9 (11.7)	
No	n (%)	96 (71.1)	33 (56.9)	63 (81.8)	
Blood transfusion	n (%)	20 (14.8)	12 (20.7)	8 (10.4)	0.095
Anemia	n (%)	20 (14.8)	12 (20.7)	8 (10.4)	0.095
Access site pseudoaneurysm	n (%)	1 (0.7)	1 (1.7)	0 (0)	0.430
Access site aneurysm	n (%)	0 (0)	0 (0)	0 (0)	----
Hematoma					
Access site	n (%)	5 (3.7)	4 (6.9)	1 (1.3)	0.278
Retroperitoneal	n (%)	2 (1.5)	1 (1.7)	1 (1.3)	----
Other	n (%)	3 (2.2)	2 (3.4)	1 (1.3)	----
No	n (%)	125 (92.6)	51 (87.9)	74 (96.1)	----
Hemoptysis	n (%)	3 (2.2)	3 (5.2)	0 (0)	0.077
Hematuria					
Gross	n (%)	4 (3)	3 (5.2)	1 (1.3)	0.364
Mild	n (%)	6 (4.4)	3 (5.2)	3 (3.9)	----
No	n (%)	125 (92.6)	52 (89.7)	73 (94.8)	----
Recurrent PE	n (%)	4 (3)	2 (3.4)	2 (2.6)	1.0
IVC filter	n (%)	9 (6.7)	5 (8.6)	4 (5.2)	0.430

Prediction of bleeding

Multivariate logistic regression analysis was done to predict bleeding. The model revealed that USCDT was associated with a 71.9% risk reduction of bleeding (OR = 0.281, 95% CI = 0.126 - 0.627, P = 0.002), controlling for age, gender, diabetes, hypertension, and obesity (Table 4).

**Table 4:** Multivariate logistic regression analysis to predict bleeding *Significant P-value; OR: Odds ratio; CI: Confidence interval; USCDT: Ultrasound-facilitated catheter-directed thrombolysis.

Parameter	OR (95% CI)	P-value
Age (years)	1.025 (0.979 - 1.073)	0.286
Gender	0.665 (0.299 - 1.481)	0.318
Diabetes	0.946 (0.36 - 2.487)	0.911
Hypertension	1.461 (0.605 - 3.53)	0.399
Obesity	0.777 (0.349 - 1.731)	0.538
USCDT	0.281 (0.126 - 0.627)	0.002*

## Discussion

The most extensively studied advanced treatment for patients suffering from acute massive and submassive pulmonary embolism is the administration of full-dose fibrinolytic therapy on a systemic level. However, over the past two decades, using this treatment has decreased, particularly among high-risk patients, due to the elevated incidence of intracranial hemorrhage associated with its use. Clinical guidelines advise against using full-dose systemic fibrinolytic therapy for treating acute submassive pulmonary embolism in all patients except those with the lowest bleeding risk [12].

In recent years, catheter-directed thrombolysis (CDT) has emerged as a promising alternative, offering targeted fibrinolytic therapy with potentially lower bleeding risks [13]. The advent of Ultrasound-Facilitated Catheter-Directed Thrombolysis (USCDT) introduces an innovative approach hypothesized to enhance the efficacy of thrombolysis by improving drug delivery to the thrombus [14]. Given the evolving landscape of APE management and the need for treatment modalities that balance efficacy with safety, our study aimed to compare the outcomes of USCDT and conventional CDT in patients with intermediate- and high-risk APE.

In our study of 135 patients treated for acute pulmonary embolism, 58 received conventional CDT, and 77 underwent USCDT, with no significant demographic or clinical differences between groups. Initial and follow-up echocardiograms, catheter assessments, and CT scans revealed no significant differences in key parameters such as TAPSE, RV/LV ratio, IVC diameter, SPAP, cardiac index, and modified Miller index between CDT and USCDT treatments, indicating comparable baseline characteristics and procedural outcomes.

Within each group, all echo parameters significantly differed at 24 hours and 90 days compared to baseline values. Regarding catheter parameters within CDT and USCDT groups, SPAP significantly differed at 12 hours and 90 days compared to baseline, and cardiac index significantly differed at 12 hours compared to baseline. Moreover, CT parameters, including RV/LV ratio and modified Miller index, significantly differed within each group at 48 hours compared to baseline.

These findings align with Sardar et al. [15], who stated a significant decrease in SPAP postprocedural compared to baseline (37.47 ±11.9 vs. 51.45 ±16). Similarly, Bagla et al. [16] revealed a decline in pulmonary artery pressure from 49.8 mmHg to 31.1 mmHg (P < 0.001). In addition, Piazza et al. [17] documented in a prospective, single-arm, multicenter trial a significant decline in SPAP in high-risk PE patients after USCDT. Kucher et al. [18] observed a significant drop in pulmonary artery pressure and a high cardiac index in the USCDT group within 24 hours. In addition, Avgerinos and colleagues reported that the mean reduction in RV/LV ratio from baseline (1.54 ± 0.30 for USCDT, 1.69 ± 0.44 for CDT) to 48 hours was 0.37 ± 0.34 in the USCDT group and 0.59 ± 0.42 in the CDT group (P = 0.01) [19].

In line, Piazza et al. [17] reported similar results regarding the RV/LV ratio and modified Miller index at 48 hours. Also, Bagla et al. [16] highlighted that the mean RV/LV ratio declined from 1.59 to 0.93 (P < 0.001), indicating a considerable improvement in RVD. Moreover, similar improvements in the RV/LV ratio and modified Miller index were reported at 48 hours in various studies [15,17,20].

In the current study, bleeding within 30 days significantly differed between patients who underwent CDT and those who underwent USCDT (P = 0.008), with major, moderate, and mild bleeding being higher in the CDT group (3.4%, 15.5%, and 24.1%, respectively) compared to the USCDT (0%, 6.5%, and 11.7%, respectively).

In a randomized study conducted by Kucher et al. on patients with intermediate-risk PE, it was found that a standardized USCDT regimen outperformed anticoagulation with heparin alone in reversing RV dilatation at 24 hours without an increased risk of bleeding complications [18]. In contrast, Rao et al. reported in their study that the rates of moderate and severe bleeding were largely identical between USCDT and CDT groups (USAT: 3%; CDT: 0%; p = 0.09) [21]. This discrepancy may be due to the relatively small sample size they included.

The outcomes of this study show the importance of technique selection in the management of APE, especially concerning bleeding risks, which remain a paramount concern in thrombolytic therapy. Our findings align with previous research suggesting that USCDT may offer advantages over conventional CDT in terms of safety, particularly by reducing the risk of bleeding complications. The mechanism behind USCDT's reduced bleeding risk could be attributed to its ability to enhance thrombolysis efficiency, allowing for lower doses of thrombolytic agents and potentially less disruption to the coagulation profile. Additionally, the targeted delivery of thrombolytic therapy may minimize systemic exposure and preserve hemostatic integrity elsewhere in the body [22,23].

Furthermore, the reduced risk of bleeding associated with USCDT has significant clinical implications, showing that USCDT could be a preferable option for patients at higher bleeding risk, such as those with a history of bleeding disorders, recent surgery, or other contraindications to aggressive anticoagulation therapy [18,24-26]. This finding is relevant in the context of intermediate to high-risk APE, where effective thrombolysis can significantly impact the patient’s outcomes, including reducing chronic pulmonary hypertension risk and improving functional status.

Multivariate logistic regression analysis was performed to quantify the impact of USCDT on bleeding risk. The analysis accounted for potential confounders, including age, gender, diabetes, hypertension, and obesity, which are known to influence bleeding risk independently. The model revealed that USCDT was associated with a 71.9% risk reduction of bleeding (OR = 0.281, 95% CI = 0.126 - 0.627, P = 0.002), revealing the safety profile of the treatment.

Limitations of our study include the relatively small number of patients. Second, the retrospective nature of this study limits the ability to control for all potential confounding factors. Therefore, prospective randomized controlled trials are needed to validate our findings.

## Conclusions

This study compared the efficacy and safety of USCDT versus conventional CDT in patients with intermediate to high-risk APE. A total of 135 patients were studied, of which 58 underwent CDT, while 77 underwent USCDT. USCDT appears to offer comparable efficacy to conventional CDT in these patients, with the added advantage of a significantly reduced risk of bleeding. Therefore, USAT has the potential to alter treatment protocols and enhance outcomes in patients with high-risk PE.

## References

[1] Harvey JJ, Huang S, Uberoi R (2022). Catheter-directed therapies for the treatment of high risk (massive) and intermediate risk (submassive) acute pulmonary embolism. Cochrane Database Syst Rev.

[2] Xia W, Yu H, Chen W, Chen B, Huang Y (2022). A radiological nomogram to predict 30-day mortality in patients with acute pulmonary embolism. Acad Radiol.

[3] Weinstein T, Deshwal H, Brosnahan SB (2021). Advanced management of intermediate-high risk pulmonary embolism. Crit Care.

[4] Epstein D, Berger G, Barda N (2018). The incidence of acute pulmonary embolism following syncope in anticoagulant-naïve patients: a retrospective cohort study. PLoS One.

[5] Meneveau N, Séronde MF, Blonde MC (2006). Management of unsuccessful thrombolysis in acute massive pulmonary embolism. Chest.

[6] Machanahalli Balakrishna A, Reddi V, Belford PM, Alvarez M, Jaber WA, Zhao DX, Vallabhajosyula S (2022). Intermediate-risk pulmonary embolism: a review of contemporary diagnosis, risk stratification and management. Medicina (Kaunas).

[7] Iaccarino A, Frati G, Schirone L (2018). Surgical embolectomy for acute massive pulmonary embolism: state of the art. J Thorac Dis.

[8] Barnes GD, Muzikansky A, Cameron S (2020). Comparison of 4 acute pulmonary embolism mortality risk scores in patients evaluated by pulmonary embolism response teams. JAMA Netw Open.

[9] Klevanets J, Starodubtsev V, Ignatenko P, Voroshilina O, Ruzankin P, Karpenko A (2017). Systemic thrombolytic therapy and catheter-directed fragmentation with local thrombolytic therapy in patients with pulmonary embolism. Ann Vasc Surg.

[10] Mostafa A, Briasoulis A, Telila T, Belgrave K, Grines C (2016). Treatment of massive or submassive acute pulmonary embolism with catheter-directed thrombolysis. Am J Cardiol.

[11] (1993). An international randomized trial comparing four thrombolytic strategies for acute myocardial infarction. N Engl J Med.

[12] Konstantinides SV, Torbicki A, Agnelli G (2014). 2014 ESC guidelines on the diagnosis and management of acute pulmonary embolism. Eur Heart J.

[13] Shah KJ, Roy TL (2022). Catheter-directed interventions for the treatment of lower extremity deep vein thrombosis. Life (Basel).

[14] Alsamman M, Choudhry AM, AlSaadi AM, Prashad R (2023). Ultrasound-accelerated catheter-directed thrombolysis. Cardiol Res.

[15] Sardar P, Piazza G, Goldhaber SZ, Liu PY, Prabhu W, Soukas P, Aronow HD (2020). Predictors of treatment response following ultrasound-facilitated catheter-directed thrombolysis for submassive and massive pulmonary embolism: a SEATTLE II Substudy. Circ Cardiovasc Interv.

[16] Bagla S, Smirniotopoulos JB, van Breda A, Sheridan MJ, Sterling KM (2015). Ultrasound-accelerated catheter-directed thrombolysis for acute submassive pulmonary embolism. J Vasc Interv Radiol.

[17] Piazza G, Hohlfelder B, Jaff MR (2015). A prospective, single-arm, multicenter trial of ultrasound-facilitated, catheter-directed, low-dose fibrinolysis for acute massive and submassive pulmonary embolism: the SEATTLE II study. JACC Cardiovasc Interv.

[18] Kucher N, Boekstegers P, Müller OJ (2014). Randomized, controlled trial of ultrasound-assisted catheter-directed thrombolysis for acute intermediate-risk pulmonary embolism. Circulation.

[19] Avgerinos ED, Jaber W, Lacomis J (2021). Randomized trial comparing standard versus ultrasound-assisted thrombolysis for submassive pulmonary embolism: the SUNSET sPE trial. JACC Cardiovasc Interv.

[20] Carroll BJ, Goldhaber SZ, Liu PY, Piazza G (2017). Ultrasound-facilitated, catheter-directed, low-dose fibrinolysis in elderly patients with pulmonary embolism: a SEATTLE II sub-analysis. Vasc Med.

[21] Rao G, Xu H, Wang JJ (2019). Ultrasound-assisted versus conventional catheter-directed thrombolysis for acute pulmonary embolism: a multicenter comparison of patient-centered outcomes. Vasc Med.

[22] Mangi MA, Rehman H, Bansal V, Zuberi O (2017). Ultrasound assisted catheter-directed thrombolysis of acute pulmonary embolism: a review of current literature. Cureus.

[23] Kline TM, Rodino AM, Dorszynski A, Murray B, Cicci J, Iyer P (2021). Ultrasound-assisted catheter-directed thrombolysis versus systemic anticoagulation alone for submassive pulmonary embolism. J Thromb Thrombolysis.

[24] Orsini S, Noris P, Bury L (2017). Bleeding risk of surgery and its prevention in patients with inherited platelet disorders. Haematologica.

[25] Burton JR, Madhavan MV, Finn M (2021). Advanced therapies for acute pulmonary embolism: a focus on catheter-based therapies and future directions. Structural Heart.

[26] Shald EA, Ohman K, Kelley D, Busey K, Erdman MJ, Smotherman C, Ferreira JA (2023). Factors associated with bleeding after ultrasound-assisted catheter-directed thrombolysis for the treatment of pulmonary embolism. Blood Coagul Fibrinolysis.

